# Effects of Feeding Varying Levels of DL-Methionine on Live Performance and Yield of Broiler Chickens

**DOI:** 10.3390/ani11102839

**Published:** 2021-09-29

**Authors:** Anthony Pokoo-Aikins, Jennifer Rumsey Timmons, Byungrok Rok Min, William Robert Lee, Samuel Njoroge Mwangi, Chongxiao Chen

**Affiliations:** 1 US National Poultry Research Center, Toxicology & Mycotoxin Research Unit, United States Department of Agriculture, Agricultural Research Services, (USDA, ARS), Athens, GA 30605, USA; 2 Food and Agricultural Science Program, Department of Agriculture, Food and Resource Sciences, University of Maryland Eastern Shore, Princess Anne, MD 21853, USA; jtimmons1@umes.edu (J.R.T.); bmin@umes.edu (B.R.M.); 3 Maple Leaf Farms, Leesburg, IN 465384, USA; billlee@mapleleaffarms.com; 4 Department of Agriculture, School of Agriculture and Applied Sciences, Alcorn State University, Lorman, MS 39096, USA; smwangi@alcorn.edu; 5 Prestage Department of Poultry Science, College of Agriculture and Life Sciences, North Carolina State University, Raleigh, NC 27695, USA; sean_chen@ncsu.edu

**Keywords:** DL-methionine, amino acid, organic broilers, meat yield

## Abstract

**Simple Summary:**

The use of DL-methionine (MET) in poultry diet formulation is vital for poultry growth because poultry do not synthesize sufficient amounts of MET needed for proper growth and performance, and currently there are insufficient natural sources of MET to fulfill the dietary needs of broiler chickens. However, the use of MET is restricted in the United States in organic poultry diets. Therefore, the objective of this study was to examine the effect of feeding different levels of dietary MET on organic broiler live performance and yield of a modern commercial broiler strain. This study gives us insight into how broiler growth and yield is affected when the allowable levels of methionine for organic broilers is further reduced, or zero methionine is used.

**Abstract:**

This study was designed to evaluate the effects of dietary supplemental DL-methionine (MET) on live performance and meat yield for broilers raised to a common weight. A total of 1552 one-day old Ross 708, sexed broilers were randomly distributed to 32 pens resulting in eight treatments (TRT) of four replicates with 44 male or 53 female/pen. A randomized complete block with a 2 × 4 (sex × 4 MET levels 0, 0.5, 1, and 2 g/kg) factorial arrangement of TRT was used. A common weight of 2400 g was approached by day 46 (1 and 2 g MET/kg feed) and day 48 (0 and 0.5 g MET/kg feed). Supplementation of MET at 1, and 2 g/kg had a lower (*p* < 0.01) feed conversion ratio (FCR) at day 46/48 than broilers fed 0.5 g MET/kg. Broilers without supplemental MET had the worst (*p* < 0.01) feed conversion and average daily gain (ADG) at day 46/48. Birds fed 0 g MET/kg of feed had lower (*p* < 0.05) whole eviscerated carcass without giblets (WOG), yield than birds fed 2 g MET/kg of feed. Additionally, birds fed 0 g MET/kg of feed had lower (*p* < 0.05) breast fillet and tender percent yields than birds fed supplemental MET. Elimination of MET from organic broiler diets resulted in reduced ADG, breast fillet yield and feed efficiency of meat yield of broilers raised to day 46/48. Reduction in MET supplementation below current levels reduced the efficiency of meat production of organic broilers raised to day 46/48.

## 1. Introduction

Methionine (MET), a sulfur containing amino acid, is considered an essential amino acid in poultry because poultry cannot naturally synthesize sufficient amounts to sustain normal body functions [1]. MET is required for buildup of the immune system and improvement of live performance, i.e., feed efficiency, muscle development and better yield [2]. Supplemental MET in broiler diets have been shown to improve body weight (BW), feed intake (FI), feed conversion ratio (FCR) and average daily gain [3]. Similar results were also reported in meat ducks [4]. MET is also considered the first limiting amino acid for poultry fed corn-soy bean-based diets [5] and therefore use of supplemental MET in poultry diets of conventional raised poultry to balance dietary amino acids (AA) is a common practice. However, synthetic AA are normally restricted or not allowed in the diet of organic raised birds [6]. In the US, MET is the only allowed synthetic AA in organic poultry feed [7], however, in October, 2012 the USDA reduced the amount of MET that can be used in organic poultry diets from 2, 2.5, and 3 kg per metric ton (4, 5, and 6 lbs per ton), to 1, 1, and 1.5 kg per metric ton (2, 2, and 3 lbs. per ton) of feed for organic laying hens, broilers, and turkeys and other poultry, respectively [7,8]. However, in a 2015 review by the National Organic Standards Board (NOSB), the average MET limit over the life of organic broilers is approximately 1.25 g MET per kg of feed (2.5 lbs per ton of feed) [9].

Diets with very limited or no amounts of synthetic MET can result in reduced performance and production which would result in low economic returns in both organic and conventional poultry production [7]. Natural alternatives to synthetic MET are allowed and encouraged by the NOSB. To this effect, the NOSB established a Methionine Task Force to develop natural alternatives for methionine supplementation for non-pastured flocks and requested periodic updates. From a 2015 review it was noted that “development of alternatives to synthetic methionine have still not reached a stage of commercial availability” [9]. The National Research Council [10], MET requirement for broiler maximum growth and feed efficiency is 0.50, 0.38 and 0.32% for broilers 0–3, 3–6 and 6–8 weeks of age, respectively. However, broilers have different requirements for different responses (maximum body weight gain, maximum feed intake, maximum breast meat yield) based on strain/breed, age and gender. However, the use of synthetic MET in the diets of organic poultry and/or broilers is banned in the European Union [7] and the possibility of eliminating supplemental MET from organic poultry diets fully in the near future in the U.S. is possible [11]. The National Organic Standards Board has come up with a step-down program to reduce and eventually eliminate the use of MET by 2040 for newer organic poultry operations [9]. Consequently, reduction or elimination of MET in organic broiler diets could increase feed costs and have a negative impact on bird health and growth [1,12], as well as have negative impact on the environment due to increased nitrogen excretion during metabolism of protein which is converted to ammonia by microbes found in poultry litter [7,13].

There is currently no single formula, resolution or feeding plan that has the potential to completely eliminate the use of non-natural MET in organic poultry [7]. Due to conflicting reports, there is the need for the organic poultry industry to start exploring how to address the MET needs of organic broilers if MET is banned entirely in the U.S.

Due to recent changes to allowable organic poultry methionine levels, it is important for the industry to continue to investigate natural methionine supplementation sources. Therefore, this study was designed to examine the effect of varying levels of dietary MET on organic broiler live performance and yield. This study is novel because it explores a treatment with no added dietary methionine to shed light on what raising organic broilers would be like without industrial sources of methionine, with primary focus on the effects on performance and yield.

## 2. Materials and Methods

The animal experiment was conducted in accordance with the recommendations and guidelines of the University of Maryland Eastern Shore Institutional Animal Care and Use Committee (AUP-Timmons-2021-4) and agreed with the standards for experiments held by the research farm.

### 2.1. Experimental Design, Birds, Diets and Housing

The study was conducted at a commercial research farm in Maryland. A total of one thousand five hundred and fifty-two (1552) sex-sorted day-old Ross 708 chicks were used in this study. The experimental design was a randomized complete block with a 2 × 4 factorial arrangement of TRTs (2 sexes × 4 MET levels) with four replicates per TRT. Birds were placed in 32 pens approximately 1 ft^2^ per bird with 16 pens containing 44 males per pen and 16 pens containing 53 females per pen. Pen density was 26.4 and 32.2 kg/sq. m for male and female chicks, respectively. Each pen was blocked based on room location and considered an experimental unit. Broilers were allocated at hatch to respective pens and fed one of the four MET diets in a three-phase feeding program (0–21, 22–35, and 36–46/48 day of age). The dietary treatments were 0 g (0%), 0.5 g (0.05%), 1.0 g (0.1%) and 2.0 g (0.2%) MET per kg of feed. Birds fed the 0 and 0.5 g MET/kg diets were raised to 48 d of age to approach a common target weight of 2.72 kg (6 lb) for all TRT. The dependent variables measured included BW, FCR, feed consumed or feed intake (FI), livability (Liv), average daily gain (ADG), yield, proximate analysis of the meat, plasma total antioxidant capacity, and relative feed efficiency. The basal corn-soybean meal diet was formulated to meet or exceed the NRC (1994) requirement for maximum growth performance, except MET.

The study was conducted in a closed-sided poultry building with a concrete floor with 48 square feet per pen. Males and females were raised in different pens. The broilers were reared in suitable pens under the same managerial, hygienic and environmental conditions. Pens were equipped with two plastic hanging feeders and automatic water drinking stations per pen. Feed and water were offered *ad libitum* and birds were raised on reused litter to a depth of five cm. The house temperature was controlled by a thermostat and maintained at 31 °C for the first week and then decreased and maintained at 27 °C to the end of the trial. The lighting program was 24 h of light and three-foot candles for days zero to seven and from day eight to processing, there were eight consecutive hours of darkness and 16 h of consecutive light using 0.75-foot candles. Similar management conditions (floor space, temperature, relative humidity, light and ventilation) were provided to all replicates.

Broiler chicks were vaccinated against Marek’s, Newcastle, infectious bronchitis and Bursal diseases. Chicks were group weighed by pen before placement. Birds were raised from day 0 to 46 or 48 depending on desired end weight. Birds were fed diets with DL-methionine (MET) (MetAMINO, 99%, Evonik Degussa GmbH, Hanau-Wolfgang, Germany). The three feed phases used in this study were the starter phase (0–21 days), the grower phase (22–35 days), and the finisher phase (36–46 or 36–48 days). Ingredients, calculated nutrients, and analyzed nutrients in the three-phase feed are presented in Table 1. Analyzed diets for the basal, treatment and control groups for the starter, grower and finisher diets are presented in Table 2. Data for body weight (BW), feed intake (FI), and mortality adjusted feed conversion ratio (FCR) were measured at the end of each feeding phase. Mortality was weighed and recorded daily. Ammonia readings and litter moisture were recorded weekly.

### 2.2. Feed and Feed Analysis

Feed samples were analyzed in the lab using the AOAC 994.12/985.28 method for amino acid profile (including, methionine, cysteine, lysine, etc.) and the AOAC 923.01 method for sulfur. Energy (calories) was analyzed using a modified Atwater calculation, consisting of carbohydrates by difference and by the following tests: Ash AOAC 942.05, fat AOAC 920.39, fiber AOAC 978.10, moisture AOAC 930.15 and protein AOAC 990.03. The basal diet was provided by a commercial poultry feed mill.

### 2.3. Litter Samples and Ammonia Readings

Approximately 0.45 kg of litter was collected from each pen weekly and analyzed for moisture and pH levels as described in (AOAC, 1990). The ammonia readings of the litter were collected inside each floor pen using an RAE LP-1200 piston hand pump and calorimetric gas detection tubes (RAE Systems, Inc., Sunnyvale, CA, USA) following the manufacturer’s protocol.

### 2.4. Blood Sample Collection

Blood samples were collected on day 45 to evaluate the effects of methionine on antioxidant capacity in live birds using oxygen radical absorbance capacity (ORAC). Two milliliters of blood were collected from the wing and vein of two broilers, randomly selected from each pen (64 samples) by venipuncture into EDTA-coated tubes. After centrifugation of the blood samples at 1500× *g* for 10 min, plasma samples were collected and stored at −80 °C for further analysis.

### 2.5. Processing

On days 46 and 48, 320 birds were randomly selected (10 birds per pen), weighed and wing-banded for processing. On day 46, the 160 birds in the TRT groups receiving 1 and 2 g of MET per kg of feed were processed while the 160 birds receiving 0 and 0.5 g of MET per kg of feed were processed on day 48.

Birds were euthanized and processed using commercial processing methods. Whole eviscerated carcasses without giblets (WOG) were drained and chilled for about 2 h in a static chiller and later stored in a walk-in refrigerator at 5 °C overnight.

### 2.6. Plasma Extraction

Plasma (200 µL) was mixed with 400 µL of ethanol and 200 µL of water for 3 min. Then, 800 µL of hexane were added and mixed for 3 min. The mixture was allowed to stand for 2 min before being centrifuged for 5 min at 14,000× *g*. The hexane layer (top layer) was removed and another 800 µL of hexane was added and mixed again for 3 min. Once again, the mixture was allowed to stand for 2 min and then centrifuged again for 5 min at 14,000× *g*. Using nitrogen gas, the remaining hexane was evaporated. The mixture was mixed with 800 µL of 0.5 M perchloric acid for 3 min before being centrifuge at 14,000× *g* for 5 min. The super natant was collected and sored at −20 °C for the total antioxidant capacity (TAC).

### 2.7. Proximate Analysis

Proximate analysis (ash, moisture, lipid, and protein) of raw breast meat samples was also conducted. Protein, ash, and moisture contents were determined using the Association of Official Analytical Chemists methods (AOAC, 2005). Lipid analysis was conducted using Folch methods described by Ahn et al. (1995) [14].

### 2.8. Carcass and Part Yield

Yield measurements were harvested manually (deboned) at a commercial deboning plant. After the WOG weight was obtained, the carcasses were cut into drumsticks, thighs, boneless thighs, wings, breast skin, deboned breast fillet, and tender. A weight was obtained for each part. The WOG yield was calculated as a percentage of the fasted live weight. The parts yield was calculated as a percentage of the chilled WOG weight.

### 2.9. Statistical Analysis

Data for all variables were analyzed by two way analysis of variance (ANOVA) with the model including the main effects of sex, methionine level and their interaction using the general linear model of Statistix version 9.0 (Analytical Software, Tallahassee, FL, USA). Tukeys all-pairwise comparison test was used to determine mean differences among treatments. Significant differences among treatments were determined at a probability of *p* < 0.05.

### 2.10. Feed Efficiency Analysis

An analysis of feed efficiency (FE) of yield parts was conducted to evaluate the efficiency of converting feed to yield meat parts. The feed efficiency was analyzed by first calculating a feed efficiency for each part by dividing the feed consumed by the mass of each “yield part” (Equation (1)).

(1) $$\mathrm{Feed}\;\mathrm{efficiency}\;\mathrm{of}\;\mathrm{each}\;\mathrm{part}=\frac{\mathrm{feed}\;\mathrm{consumed}\;\left(\mathrm{kg}\right)}{\mathrm{mass}\;\mathrm{of}\;“\mathrm{yield}\;\mathrm{part}”\;\left(\mathrm{kg}\right)}$$

The mass of each “yield part” is calculated by multiplying the body weight of birds from day 46/48 MET Level main effect by the percent yields of each “yield part.” (See Equation (2))

(2) Mass of yield part (kg)=(body weight (kg)×percent yield of yield part)/100

The feed efficiency of each part is then compared relative to feed efficiency at the standard treatment of 1 g MET/kg feed (FER). The comparison of feed efficiencies is calculated first by calculating the average feed efficiency for each part at 1 g MET/kg. The average feed efficiency at 1 g MET/kg feed for each part is then divided by the feed efficiency of each part and multiplied by 100 to give a percent relative feed efficiency (Equation (3)).

(3) $$\mathrm{Percent}\;\mathrm{relative}\;\mathrm{feed}\;\mathrm{efficiency}\;\left(\%\right)=\frac{\mathrm{average}\;\mathrm{feed}\;\mathrm{efficiency}\;\mathrm{of}\;\mathrm{each}\;\mathrm{yield}\;\mathrm{part}\;\mathrm{at}1\;\frac{g\;\mathrm{MET}}{\mathrm{kg}\;\mathrm{feed}}}{\mathrm{average}\;\mathrm{feed}\;\mathrm{efficiency}\;\mathrm{of}\;\mathrm{each}\;“\mathrm{yield}\;\mathrm{part}”}$$

where a “yield part” is, for example drumstick, thigh, boneless thigh, wing, breast skin, breast fillet, or tender. When the percent relative feed efficiency is at 100, the MET level leads to a feed efficiency that is comparable the standard of 1 g MET/kg feed. When the percent relative feed efficiency is below 100, the MET level leads to a feed efficiency that is below the standard of 1 g MET/kg feed. When the percent relative feed efficiency is above 100, the MET level leads to a feed efficiency that is above the standard of 1 g MET/kg feed. 

## 3. Results and Discussions

### 3.1. Live Performance

Broilers have differing requirements for growth, performance and yield depending on age, breed, and gender. Ross broiler nutrition specifications for conventional broilers raised to 2.50–3.00 kg target live weight states the total methionine requirement for maximum weight gain, feed and feed efficiency are 0.56, 0.51, 0.47, and 0.44% for starter grower, finisher one and finisher two, respectively. Additionally, the total methionine + cysteine requirements for Ross conventional broilers are 1.08, 0.99, 0.90, and 0.85%, respectively, for starter grower, finisher one and finisher two. In this study the focus was on organic broiler nutritional requirements, which are much lower than the requirements for Ross broilers for growth, performance and meat yield in terms of methionine. The live performance results are provided in Table 3. There were no significant (*p* > 0.05) interactions between sex × MET level on live performance parameters for all phases. Supplementing MET to the diets promoted bird growth. Birds fed diets with 0 g supplemental Met/kg had lower (*p* < 0.05) BW when compared to birds fed diets with supplemental MET levels at day 21 and 35 and when compared with birds fed 0.5 g MET/kg at day 46/48. These results demonstrate that supplemental MET contributes to body weight gain. MET helps with metabolism and in synthesizing the proteins that contribute to muscle, organ and feather formation, all which contribute to BW.

In an experiment by Chattopadhyay et al. (2006), they found that broilers fed 10 g of DL-MET/kg diet supplementation for 0–21 and 0–42 days had significantly higher BW than the control group for the same periods. The group with 10 g of herbal MET/kg diet supplementation had no significant increase in BW, while when herbal MET supplementation was increased to 15 g/kg diet there was significant improvement in BW for day 0–21 and day 0–42 compared with the control. These results demonstrate that lower levels of DL-MET than natural MET sources (herbal methionine) are required to improve the BW of broilers. The results of the current study suggests that by day 46/68, feeding higher levels of DL-MET resulted in improved body weight.

Majdeddin, et al. (2019) reported that increasing the levels of MET with cysteine levels the same across all groups (low (0.4 g/kg below total sulfur amino acid (TSAA) requirements), medium (at TSAA requirement), and high (0.4 g/kg above TSAA requirements)) improved growth [3]. Agostini et al. (2016) reported that diets supplemented with MET resulted in higher (*p* < 0.001) BWG, FI, and better FCR compared to a basal diet with 0 MET supplementation [15]. Thirty percent extra DL-MET (a synthetic methionine with 99% methionine availability) in grower and finisher broilers diets resulted in a positive effect on growth [16].

Rubin et al. (2007) supplemented a broiler basal diet with 0.31, 0.51%, and 0.66% MET at day 21 and found that birds fed the lowest MET level had significantly less BW than the birds fed the two higher levels of MET [17]. Once again, the birds fed the lowest MET level had significantly lower BW than the birds fed the two higher levels of MET.

Mulyantini et al. (2010) indicated that an increase in digestible MET (from 3 g/kg diet to 4.5, 6, 7.5, and 9 g/kg diet) significantly increased the BW of birds at 21 days of age [18]. Ahmed and Abbas, (2011) looked at the effects of dietary levels of MET on broiler performance and carcass characteristics for broiler diets with four levels of dietary MET in the starter diet and the finisher diet [19]. The addition of MET significantly maximized BW gain at day 21, 35, and for birds fed 0.5 g MET/kg at day 46/48. Additionally, MET supplementation increased the end of trial ADG. The results of experiments done by other researchers and the results of the current experiment suggest that MET is required to promote BW in broilers.

Apart from supplemental MET, reduced crude protein in broiler diets may result in negative growth performance [20]. The crude protein level in the starter basal diet was high, which resulted in high crude protein level in all the starter diets for all the treatments.

In this study, the sex of the broilers had an effect on BW. Male broilers had higher BW (*p* < 0.01) than female broiler throughout the trial (Table 3). Male broilers being larger than female broilers is a well-known trend in the broiler industry. Male broilers tend to have superior growth rates and are more efficient at converting feed into muscle. The ratio of male to female BW in this study was 1.23:1 (2641 g vs. 2144 g, respectively) at day 46/48.

The result of the present study agrees with findings by Hernández et al. (2012) in a study where the weight gain (WG) of male broilers was higher (*p* < 0.05) than the WG of females from placement to day 42 [21].

From day 0–21, MET levels did not have any significant effect on feed efficiency. Contrary to the results in the present experiment, Mulyantini et al. (2010) indicated that increasing digestible MET levels significantly improved the FCR of birds at 21 days of age [18]. A reduced feed efficiency is common with unbalanced diets or diets that are deficient in a specific nutrient [22].

At day 35, there was a significant difference in feed efficiency between broilers fed 0 g MET/kg of feed and those fed 2 g MET/kg of feed (*p* < 0.05), indicating that 2 g MET/kg of feed in the diet enabled the broilers to utilize their feed more efficiently than birds fed 0 g MET/kg of feed. Furthermore, the results seem to suggest that the birds should be fed different levels of MET at different phases.

The birds fed the 0 g MET/kg diet had higher (*p* ≤ 0.05) FCR than birds fed 2 g MET/kg diet at day 35, and at day 46/48, birds fed the 0 g MET/kg diet had the highest FCR and birds fed the 1, and 2 g MET/kg diets led to lower (*p* < 0.01) FCR than the FCR of broilers fed 0.5 g MET/kg. Other studies have exhibited the same trend of improved FCR with increased MET supplementation beyond day 21. Several researchers including Halder and Roy, (2007), Rubin et al. (2007) and Ahmed and Abbas, (2011) have reported improved FCR with MET supplementation [17,19,23]. Golshahi et al. (2013) reported the same trend with FCR and MET levels in a trial where broilers were fed starter, grower and finisher diets with increasing MET levels and varied MET levels at each phase [24].

In this trial, although the effects of MET supplementation on FCR were significant at day 35 and at day 46/48, the effects of sex on FCR were only significant at day 46/48 since male broilers had lower (*p* < 0.05) FCR compared with the FCR of female broilers (Table 3). A review of the literature indicates that male birds will have a lower FCR compared with the FCR of female broilers due to their higher genetic potential for BW [5,25,26,27,28]. FCR increased with increasing age of birds in both sexes [25].

In this study, by day 46/48, birds fed 0.5 gMET/kg diet had higher (*p* < 0.01) FI than birds fed 1 and 2 g MET/kg diets. Ribeiro et al. (2005) reported no change in FI due to MET supplementation [29]. Some studies have reported that an increase in digestible MET significantly increased FI [17,18]. Still other studies have reported that birds fed intermediate levels (between the control (no MET) and the highest levels) of MET supplementation had significantly higher FI than birds fed diets with no MET or the highest levels of MET [23]. Other studies have shown that high levels of MET supplementation have led to decreased broiler FI [18,22]. Also, Carew et al. (1998) fed a diet with 3.84 times more MET than the NRC requirement for growth and feed efficiency from day 10 to 24 of age and observed 16% reduction in FI [30].

There were significant differences in FI based on sex (Table 3). Male broilers consumed more feed (*p* < 0.01) than female broilers throughout all the growth phases. The results from this trial are in agreement with studies by Taha et al. (2011). These researchers reported that male broilers of three strains consumed significantly more feed than the females [31]. Male broilers consumed more feed yet had a better FCR by the end of the trial than female broilers in this study.

### 3.2. Litter Quality

The litter moisture from the pens fed the 1 g MET/kg dietary TRT was *p* < 0.05 less than the percent moisture of the litter from pens raised with birds fed the 0 g MET dietary TRT at day 21. The litter moistures from the 0.5 g and 2 g MET groups were not significantly different from 0 g MET (Table 4).

At day 35, broilers fed 2 g/kg of feed had significantly higher (*p* < 0.01) litter percent moisture than birds fed either 0.5 or 1 g of MET/kg of feed (Table 4). These results suggest that as the experiment progressed, the birds fed 2 g MET/kg of feed indeed had an excess of MET in the feed and the effects of excess MET in the feed became more evident by this phase. The analyzed starter and grower diet for the 2 g MET/kg TRT showed that the percent MET levels exceeded the NRC recommended levels of MET.

Khajali et al. (2006) observed no significant difference in the litter moisture at day 42 [32], which is in agreement with the observation in this study where we saw no significant differences in litter moisture at day 46/48 (Table 4). The impact of added methionine on litter moisture is that it could reduce overall crude protein through soybean meal which would reduce litter moisture.

### 3.3. Total Antioxidant Capacity and Proximate Analysis

There were no significant effects of MET levels or sex and there were no significant interactions on the contents of ash, moisture, lipid and protein in breast meat (Table 5). There were also no significant effects of MET or sex and no significant interaction on plasma TAC (Table 5).

### 3.4. Yield

MET has been reported to improve performance as well as increase meat yield [2,12,33,34]. The different dietary MET levels fed to broiler chickens had an effect on the percent WOG yield. There was a significant difference (*p* < 0.01) between broilers fed 2 g of MET and 0 g of MET/kg of feed. In this study, the highest level of supplemental MET increased the WOG yield. It was anticipated that there would be an increase in WOG yield with increasing MET supplementation and that the WOG of male broilers would be higher for all treatments. It may be difficult to predict or note differences in WOG. The breast fillet is of more significance because it is directly tied to muscle accretion. The WOG includes other parts of the broilers (besides the breast fillet) for which there may be various factors at play with regards to growth, development and metabolism of those parts.

In contrast, Corzo et al. (2005) studied the effects of AA density on carcass trait yields at day 56 and found no significant effect of diet on WOG percentage [35]. Although in this study there were differences in WOG yield due to MET level, there was no difference between the WOG of males and females. These results are similar to those reported by Corzo et al. (2005) and Qudsieh, (2014) [35,36].

The percentage of breast fillet from birds fed the 0 g MET/kg TRT was significantly lower compared to the percentage of breast fillet from birds fed the 0.5, 1 and 2 g MET/kg (Table 6). There were no differences between the 0.5 and 1 g MET/kg TRT but the 2 g MET/kg TRT was higher (*p* < 0.05). The results of the current research reveal that the percent breast fillet was affected by feeding different levels of DL-MET. There was a direct relationship between the breast fillet percentage and MET since as MET level increased, breast fillet percentage also increased. In short, MET supplementation improved breast meat yield.

Supplemental MET increases breast muscle [37]. Increasing dietary MET level in broilers above the NRC (1994) recommended levels resulted in significant improvement of broiler breast muscle development [38]. Ojano-Dirain and Waldroup (2002) observed a significant improvement in dressing percentage and breast meat yield between broilers fed NRC MET level, and those fed higher levels [39].

Schutte and Pack (1995a) reported that the requirement for TSAA, including MET for high breast meat yield, is greater than the requirement for high BW [34]. Based on the present study, increasing the dietary MET level from 1 g to 2 g significantly increased the percent of breast by 1.02%, which suggests that increasing digestible MET could improve meat yield (Table 6).

Female broilers had a significantly higher (*p* < 0.01) breast meat fillet percentage compared to the breast meat percentage from male broilers (Table 5), although male broilers had higher BW compared to the BW of female broilers. This finding agrees with the other literature sources [40,41].

The tender percentages from the broilers fed the three highest levels of supplemental MET were significantly higher (*p* < 0.01) compared to the tender percentage from broilers fed 0 g MET/kg of feed (3.81%) (Table 5). Supplemental MET improved tender yield although there was no direct trend as was seen with the breast fillet (i.e., the tender yield did not increase with increasing MET levels). Since the tender is a smaller portion of meat than the breast fillet, there may not have been enough tender to cause the sensitivity in the data that was seen with the breast fillet. It was expected that supplementation with MET would benefit the tender yield since the tender is closely associated with the breast fillet. Albrecht et al. (2019) reported that fillet weight was significantly correlated to the MET concentration (*p* < 0.001) in the diets, and it was also reflected in the significant (*p* < 0.001) difference in weight gain between concentration groups [42].

In this study, male broilers also yielded more (*p* < 0.01) percentage of drumsticks than female broilers (Table 6). Females also yielded significantly higher percentages of wings than male broilers. These results suggest that male broilers yielded more dark meat and female broilers yielded more boneless skinless white meat including breast fillet, tenders, and wings. This finding is in agreement with results obtained by Corzo et al. (2005) which indicated that male broilers yielded significantly more drumsticks compared with female broilers at days 42 and 56 and that although female broilers yielded a larger proportion of tenders, male broilers might have compensated by yielding more drumsticks [35]. Raising broiler males and females separately may result in different feeding strategies to maximize yield, especially if there is a market for a specific part.

### 3.5. Feed Efficiency of Meat Production

Calculations were made based on feed conversion to meat to demonstrate the relationship between supplemental MET level and feed efficiency (Table 7 and Table 8). The feed efficiency calculations were based on yield and on the equations presented in Section 2.10. This relationship between MET level and feed efficiency of meat is a measure of the relative effects of MET supplementation level on the cost of production. 

From the information presented in Table 7, it is evident that MET plays a major role in the efficiency of chicken meat yield. Per our calculations (Table 7 and Table 8), when MET levels were fed at the 1 g MET/kg feed (standard), those that were fed MET levels below the standard had decreased feed efficiency of yield parts and those fed MET levels above the standard had increased feed efficiency based on yield parts. 

The relative impact of cost can be seen with the results of the feed efficiency calculation. Rostagno et al. (1995) found that there were considerable benefits for feed cost per kg broiler weight or per kg breast weight when birds were fed a diet supplemented with digestible DL-MET [43]. The FE and FER results could be attributed to the fact that muscle accretion was less for birds fed no additional MET, since MET helps with muscle accretion. Feed efficiency of meat yield is directly related to both the monetary and environmental costs of production. The better the feed efficiency of meat yield, the less feed is required to produce the final consumer product. From this perspective, further reduction in supplemental methionine will result in further deterioration in the both the affordability and environmental sustainability of organically produced chicken meat.

## 4. Conclusions

In conclusion, it took two extra days for birds fed 0 and 0.5 g of MET/kg of diet to attain a weight closer to those fed 1 and 2 g of supplemental MET/kg of diet. This shows that if the USDA further reduces the synthetic organic broiler MET requirement for growth and yield, more time will be needed for raising the birds, leading to extra labor and production costs. These added costs will be passed on to the consumer. Birds fed diets with supplemental DL-MET level had better results in FCR at the end of the trial. MET supplementation increased the ADG. Birds fed 0.5 g/kg had higher BW than birds fed diets with no added MET. These performance results show the important role that methionine plays in organic broiler diets. As a result, the use of DL-MET could increase muscle accretion resulting in more yield. Methionine has an immune function and can affect feathering and many other metabolic functions, and it has many potential effects on broiler health. Efficiency is sacrificed with the elimination of supplemental levels of DL-MET, which can lead to increased monetary and environmental costs of organic meat production. Agencies should critically review the overall benefits of DL-MET when making decisions, especially the decision to eliminate industrial sources of MET. This research is necessary because it demonstrates how industrial methionine is essential to the poultry industry, contributing to producing broiler meat at optimum efficiency.

## Figures and Tables

**Table 1:** Composition of the basal diets in 3-phases.

Ingredient	Day 0–21 (Starter)	Day 21–35 (Grower)	Day35–46/48 (Finisher)
Corn	47.78	53.96	60.66
Soybean Meal	46.75	39.80	33.35
Filler ^1^	0.20	0.20	0.20
Vegetable Oil	2.55	3.40	3.75
Defluorinated Phosphate	1.96	1.64	1.07
Limestone	0.44	0.51	0.51
Salt	0.15	0.21	0.29
Betaine 32%	0.00	0.09	0.05
Trace minerals ^2^	0.05	0.06	0.06
DL-methionine	0.00	0.00	0.00
Vitamin Premix ^3^	0.05	0.05	0.03
Enzyme ^4^	0.05	0.05	0.00
Calsporin ^5^	0.02	0.03	0.03
Avg. Cost ($/kg)	0.45	0.43	0.40
Calculated nutrients ^6^ (Analyzed nutrients)			
Crude Protein (%)	26.19 (25.83)	23.28 (22.99)	20.62 (20.50)
Moisture (%)	NA (12.52)	NA (10.92)	NA (12.79)
Fat (Crude) (%)	4.50 (4.99)	5.44 (5.86)	5.91 (6.38)
Fiber (Crude) (%)	NA (2.87)	NA (2.42)	NA (1.96)
Ash (%)	5.66 (5.98)	5.19 (5.52)	4.46 (4.64)
TMEn ^7^ (Kcal/kg)	3022 (3002)	3132 (3133)	3220 (3140)
Sulfur (%) ^8^	NA (0.23)	NA (0.22)	NA (0.193)
Met (%) ^9^	NA (0.31)	NA (0.32)	NA (0.27)
Cys (%) ^10^	NA (0.38)	NA (0.31)	NA (0.31)
Lys (%) ^11^	NA (1.43)	NA (1.42)	NA (1.17)

^1^ Filler: Sand; the filler levels were varied in different treatments (0 g MET/kg of feed had 0% added MET and 0.2% filler, 0.5 g MET/kg had 0.05% added MET and 0.15% filler, 1 g MET/kg of feed had 0.10% added MET in the feed and 0.10% filler, 2 g MET/kg of feed, had 0.20% MET in the feed and 0% filler). ^2^ Mineral premix per kilogram of diet: 0.264 mg Se as Na_2_SeO_3_; 12.80 mg Cu as CuSO_4_.5H_2_O; 0.24 mg I as KIO_3_; 106.67 mg Fe as FeSO_4_-H_2_O; 81.36 mg Mn as MnSO_4_-H_2_O. Determined by analysis of duplicate samples. ^3^ Vitamin premix per kilogram of diet: 11,025 I.U. vitamin A, 3528 I.U. vitamin D3, 33 I.U. vitamin E, 0.91 mg vitamin K, 2 mg thiamin, 8 mg riboflavin, 55 mg niacin, 18 mg Ca pantothenate, 5 mg vitamin B. ^4^ Enzyme refers to endo-1,3(4)-beta-glucanase and endo-1,4-beta-xylanase, used as a feed additive for chickens. ^5^ Calsporin refers to *Bacillus subtilis* C-3102, probiotic that is beneficial to gut health. ^6^ NA indicates that data for this variable (calculated nutrient) is not available. ^7^ TMEn = Total Metabolizable Energy. ^8^ The analyzed sulfur (%) for 0, 0.5, 1, and 2 g MET/kg feed for the started diets are 0.23, 0.24, 0.25 and 0.27, respectively; for the grower diets are 0.20, 0.22, 0.23 and 0.25, respectively; and for the finisher diets are 0.197, 0.196, 0.14 and 0.238, respectively. ^9^ The analyzed Met (%) for 0, 0.5, 1, and 2 g MET/kg feed for the started diets are 0.37, 0.40, 0.44 and 0.53, respectively; for the grower diets are 0.31, 0.38, 0.39 and 0.47, respectively; and for the finisher diets are 0.33, 0.33, 0.39 and 0.43, respectively. ^10^ The analyzed Cys (%) for 0, 0.5, 1, and 2 g MET/kg feed for the started diets are 0.32, 0.42, 0.40 and 0.39, respectively; for the grower diets are 0.36, 0.32, 0.32 and 0.28, respectively; and for the finisher diets are 0.26, 0.30, 0.28 and 0.31, respectively. ^11^ The analyzed Lys (%) for 0, 0.5, 1, and 2 g MET/kg feed for the started diets are 1.46, 1.45, 1.48 and 1.47, respectively; for the grower diets are 1.42, 1.41, 1.41 and 1.30, respectively; and for the finisher diets are 1.21, 1.26, 1.28 and 1.27 respectively.

**Table 2:** Analyzed starter (Str), grower (Gr), and finisher (Fin) diets and MET dietary treatments.

Feed Ingredients	0 g MET/kg			0.5 g MET/kg			1.0 g MET/kg			2.0 g MET/kg		
	Str	Gr	Fin	Str	Gr	Fin	Str	Gr	Fin	Str	Gr	Fin
Crude Protein (%)	26.49	22.2	20.57	26.09	22.89	21.13	25.61	22.24	21.11	25.54	23.73	20.36
Moisture (%)	12.38	12.62	12.44	12.16	12.83	12.42	12.31	12.75	12.52	12.45	12.68	12.58
Fat (Crude) (%)	4.9	5.88	6.26	4.77	5.75	6.41	5.07	5.78	6.32	4.74	5.90	6.45
Fiber (Crude) (%)	2.58	2.56	2.24	2.90	3.05	1.88	2.85	2.22	2.31	2.995	2.33	2.22
Ash (%)	6.015	5.35	4.80	6.03	5.35	4.86	6.02	5.46	4.03	5.97	5.18	4.54
Calories Kcal/kg	3011	3075	3131	3000.5	3044	3150	3012.5	3074	3156	2987.5	3088	3146
Sulfur (%)	0.23	0.20	0.197	0.24	0.22	0.196	0.25	0.23	0.214	0.27	0.25	0.238
Met (%)	0.37	0.31	0.33	0.40	0.38	0.33	0.44	0.39	0.39	0.53	0.47	0.43
Cys (%)	0.32	0.36	0.26	0.42	0.32	0.30	0.40	0.32	0.28	0.39	0.28	0.31
Lys (%)	1.46	1.42	1.21	1.45	1.41	1.26	1.48	1.41	1.28	1.47	1.30	1.27

**Table 3:** The effects of DL-methionine levels on live production performance of broilers at day 21, day 35 and day 46/48 ^1^.

Treatment	Day 0–21				Day 0–35				Day 0–46/48				
	Feed Intake (g)	FCR (g:g)	Body Weight (g)	Liv (%)	Feed Intake (g)	FCR (g:g)	Body Weight (g)	Liv (%)	Feed Intake (g)	FCR (g:g)	Body Weight (g)	Liv (%)	Average Daily Gain (g/Day)
Main Effect													
Sex													
Male	896.9 ^a^	1.40	638.2 ^a^	96.6	2603 ^a^	1.61	1614 ^a^	95.64	4750 ^a^	1.78 ^b^	2641 ^a^	94.60	56.22 ^a^
Female	800.1 ^b^	1.39	575.2 ^b^	96.2	2244 ^b^	1.64	1369 ^b^	96.06	3971 ^b^	1.85 ^a^	2144 ^b^	95.87	45.66 ^b^
SEM	18.13	0.029	4.69	0.0069	23.35	0.014	8.76	0.0078	39.88	0.0099	24.59	0.82	0.52
Main Effect													
MET Level													
0.0 g	831.5	1.45	574.8 ^b^	96.4	2388	1.68 ^a^	1418 ^c^	95.56	4424 ^a,b^	1.91 ^a^	2311 ^b^	95.37	48.1 ^b^
0.5 g	812.7	1.34	605.5 ^a^	97.8	2397	1.61 ^a,b^	1492 ^b^	97.50	4593 ^a^	1.83 ^b^	2483 ^a^	96.03	51.7 ^a^
1.0 g	863.1	1.40	615.5 ^a^	94.9	2438	1.61 ^a,b^	1514 ^a,b^	94.33	4203 ^b^	1.77 ^c^	2369 ^a,b^	93.81	51.5 ^a^
2.0 g	886.6	1.40	631.3 ^a^	96.5	2470	1.60 ^b^	1542 ^a^	96.01	4224 ^b^	1.75 ^c^	2410 ^a,b^	95.74	52.4 ^a^
SEM	25.64	0.042	6.63	0.0098	33.03	0.019	12.39	0.011	56.39	0.014	34.77	1.16	0.73
Interaction													
MET Level × Sex													
0.0 g/Male	864.8	1.44	601.0	97.4	2525	1.68	1533	96.22	4917	1.89	2584	95.46	53.84
0.5 g/Male	893.4	1.40	637.8	97.5	2602	1.60	1619	96.88	4973	1.80	2716	94.89	56.59
1.0 g/Male	891.8	1.38	644.6	95.5	2590	1.59	1629	94.32	4531	1.73	2600	93.75	56.51
2.0 g/Male	937.6	1.40	700.0	96.1	2650	1.58	1676	95.15	4582	1.71	2665	94.32	57.93
0.0 g/Female	798.2	1.45	548.4	95.4	2206	1.69	1303	94.90	3931	1.93	2037	95.28	42.44
0.5 g/Female	732.0	1.28	572.9	98.1	2193	1.61	1364	98.11	4213	1.86	2250	97.17	46.89
1.0 g/Female	834.6	1.42	586.5	94.3	2287	1.64	1399	94.34	3875	1.80	2138	93.87	46.47
2.0 g/Female	835.5	1.41	592.8	96.9	2291	1.63	1408	96.88	3866	1.79	2154	97.17	46.83
SEM	36.27	0.059	9.38	0.014	46.71	0.028	17.52	0.016	79.75	0.014	49.18	1.64	1.04
ANOVA	*p*-Value												
Sex	<0.01	0.77	<0.01	0.68	<0.01	0.17	<0.01	0.71	<0.01	0.01	<0.01	0.28	<0.01
MET Level	0.21	0.38	<0.01	0.26	0.29	0.02	<0.01	0.27	<0.01	<0.01	<0.01	0.54	<0.01
MET Level × Sex	0.48	0.52	0.61	0.70	0.73	0.82	0.61	0.77	0.21	0.79	0.80	0.73	0.82

^a,b,c^ Means within a column with different superscripts differ significantly by *p* < 0.05. MET source: DL-MET. 0.0 g = Broilers fed diet with 0 g per kg of feed added MET; 0.5 g = Broilers fed diet with 0.5 g per kg of feed added MET; 1 g = Broilers fed diet with 1 g per kg of feed added MET; 2 g = Broilers fed diet with 2 g per kg of feed added MET. Livability = Liv; Day 46 or 48; Birds fed diets with 0 and 0.5 g MET per kg of feed were raised to day 48. Birds fed diets with 1 g and 2 g MET per kg o+f feed were raised to day 46. ^1^ Data are means of four replicate pens with either 44 males per pen or 53 females per pen.

**Table 4:** The effects of DL-methionine levels on litter quality of broilers at day 21 and day 35 ^1^.

Treatment	Day 0–21			Day 0–35			Day 0–46/48	
	Litter Moisture (%)	Litter pH	Litter Moisture (%)	Litter pH	Ammonia (ppm)	Litter Moisture (%)	Litter pH	Ammonia (ppm)
Main Effect								
Sex								
Male	26.61	7.8	25.66 ^a^	8.7	10.3	26.94	8.6	11.94
Female	23.50	7.6	23.04 ^b^	8.6	10.3	26.29	8.5	11.19
SEM	1.26	0.139	0.80	0.036	1.189	0.76	0.027	1.08
Main Effect								
MET Level								
0.0 g	27.85 ^a^	7.7	26.16 ^a,b^	8.6	11.88	26.47	8.6	12.12
0.5 g	24.58 ^a,b^	7.8	22.43 ^b^	8.6	9.63	26.19	8.5	11.38
1.0 g	20.58 ^b^	7.6	21.78 ^b^	8.6	10.75	27.01	8.6	11.00
2.0 g	27.22 ^a,b^	7.5	27.02 ^a^	8.6	8.75	26.78	8.5	11.75
SEM	1.78	0.18	1.14	0.052	1.67	1.08	0.038	1.52
Interaction								
Met Level × Sex								
0.0 g/Male	31.13	8.0	26.95	8.7	12.8	27.88	8.6	10.0
0.5 g/Male	26.22	8.0	23.16	8.6	9.8	25.29	8.6	12.0
1.0 g/Male	19.87	7.7	22.42	8.7	9.0	26.61	8.5	12.0
2.0 g/Male	29.24	7.5	30.08	8.6	9.5	27.99	8.5	14.0
0.0 g/Female	24.56	7.5	25.37	8.6	11.0	25.07	8.5	14.2
0.5 g/Female	22.94	7.7	21.70	8.6	9.5	27.10	8.4	10.8
1.0 g/Female	21.29	7.5	21.14	8.6	12.5	27.42	8.6	11.0
2.0 g/Female	25.20	7.5	23.96	8.5	8.0	25.58	8.5	8.75
SEM	2.52	0.26	1.61	0.074	2.36	1.53	0.054	2.15
ANOVA	*p*-Value							
Sex	0.095	0.22	0.03	0.24	1.00	0.55	0.09	0.63
MET Level	0.035	0.54	<0.01	0.59	0.58	0.95	0.41	0.96
MET Level × Sex	0.47	0.78	0.39	0.85	0.67	0.36	0.07	0.16

^a, b^ Means within a column with different superscripts differ significantly by *p* < 0.05. MET source: DL-MET. 0.0 g = Broilers fed diet with 0 g per kg of feed added MET; 0.5 g = Broilers fed diet with 0.5 g per kg of feed added MET; 1 g = Broilers fed diet with 1 g per kg of feed added MET; 2 g = Broilers fed diet with 2 g per kg of feed added MET. Day 46 or 48; Birds fed diets with 0 and 0.5 g MET per kg of feed were raised to day 48. Birds fed diets with 1 g and 2 g MET per kg of feed were raised to day 46. ^1^ Data are means of four replicate pens with either 44 males per pen or 53 females per pen.

**Table 5:** The effects of feeding added DL-methionine level on Proximate Analysis of meat and TAC of broilers ^1^.

Treatment	Ash (g)	Moisture (%)	Lipid (g)	Protein (%)	Plasma TAC (mmol TE/L Plasma)
Main Effect					
Sex					
Male	0.91	84.22	0.71	25.53	786.14
Female	0.90	85.36	0.65	25.59	754.74
SEM	0.043	0.89	0.033	0.19	44.57
Main Effect					
MET Level					
0.0 g	0.86	85.13	0.68	25.20	760.43
0.5 g	0.98	84.50	0.72	25.41	668.72
1.0 g	0.95	84.68	0.58	25.73	785.00
2.0 g	0.84	84.86	0.74	25.92	867.61
SEM	0.061	1.25	0.046	0.28	63.03
Interaction					
MET Level × Sex					
0.0 g/Male	0.82	85.15	0.73	25.52	838.46
0.5 g/Male	0.98	84.13	0.73	25.12	644.89
1.0 g/Male	1.00	84.95	0.57	26.12	791.80
2.0 g/Male	0.86	82.67	0.74	25.34	869.39
0.0 g/Female	0.89	85.11	0.63	24.88	682.39
0.5 g/Female	0.98	84.85	0.72	25.70	692.56
1.0 g/Female	0.91	84.42	0.59	25.30	778.19
2.0 g/Female	0.82	87.05	0.64	26.48	865.82
SEM	0.087	1.78	0.065	0.39	89.18
ANOVA			*p*-Value		
Sex	0.79	0.38	0.15	0.84	0.62
MET Level	0.31	0.99	0.09	0.29	0.20
MET Level × Sex	0.81	0.52	0.35	0.052	0.70

Notes: Means within a column with different superscripts differ significantly by *p* < 0.05. MET source: DL-MET. 0.0 g = Broilers fed diet with 0 g added MET per kg of feed; 0.5 g = Broilers fed diet with 0.5 g added MET per kg of feed; 1 g = Broilers fed diet with 1 g added MET per kg of feed; 2 g = Broilers fed diet with 2 g added MET per kg of feed. ^1^ Data are means of four replicates of two birds per pen.

**Table 6:** The effects of sex and DL-methionine levels on yield carcass (WOG, wings, breast skin, breast fillet, and tender) of broilers ^1^.

Treatment	WOG Yield (%)	Wings (%)	Breast Skin (%)	Breast Fillet (%)	Tender (%)	Avg Live Wt. (g)	Drumsticks (%)	Thighs (%)	Boneless Thighs (%)
Main Effect									
Sex									
Male	71.56	7.84 ^b^	2.33 ^b^	17.33 ^b^	3.83 ^b^	2828 ^a^	10.38 ^a^	14.03	10.95 ^a^
Female	72.51	8.15 ^a^	2.59 ^a^	18.23 ^a^	4.31 ^a^	2273 ^b^	9.69 ^b^	13.86	10.48 ^b^
SEM	0.39	0.089	0.036	0.18	0.44	20.34	0.16	0.09	0.079
Main Effect									
MET Level									
0.0 g	70.66 ^b^	7.85	2.48	15.95 ^c^	3.81 ^b^	2455 ^b^	10.11	14.28	10.88
0.5 g	71.41 ^a,b^	7.99	2.41	17.76 ^b^	4.15 ^a^	2679 ^a^	10.31	13.83	10.66
1.0 g	72.81 ^a,b^	8.01	2.50	18.19 ^b^	4.15 ^a^	2528 ^b^	9.94	14.00	10.70
2.0 g	73.24 ^a^	8.15	2.44	19.21 ^a^	4.16 ^a^	2540 ^b^	9.76	13.68	10.61
SEM	0.56	0.13	0.050	0.25	0.06	28.77	0.22	0.15	0.11
Interaction									
Met Level × Sex									
0.0 g/Male	69.90	7.75	2.33	15.15	3.57	2727	10.40	14.38	11.18
0.5 g/Male	70.62	7.65	2.33	17.20	3.88	2994	10.85	13.70	10.76
1.0 g/Male	71.75	7.95	2.30	17.60	3.90	2811	10.18	14.28	10.98
2.0 g/Male	73.98	8.03	2.35	19.38	3.98	2782	10.08	13.78	10.93
0.0 g/Female	71.43	7.95	2.63	16.75	4.05	2183	9.83	14.18	10.58
0.5 g/Female	72.20	8.33	2.50	18.33	4.43	2364	9.78	13.95	10.60
1.0 g/Female	73.86	8.08	2.70	18.78	4.40	2246	9.70	13.76	10.43
2.0 g/Female	72.50	8.28	2.53	19.05	4.35	2299	9.45	13.58	10.30
SEM	0.79	0.18	0.071	0.35	0.089	40.69	0.32	0.22	0.16
ANOVA	*p*-Value								
Sex	0.11	0.02	<0.01	<0.01	<0.01	<0.01	<0.01	0.27	<0.01
MET Level	0.01	0.43	0.62	<0.01	<0.01	<0.01	0.36	0.06	0.40
MET Level × Sex	0.13	0.43	0.34	0.06	0.79	0.373	0.79	0.36	0.37

^a,b,c^ Means within a column with different superscripts differ significantly by *p* < 0.05. WOG = Whole eviscerated carcass without giblets (without feathers, neck, head, viscerals, tail, and feet). WOG is a percentage of average live weight. The other parts are a percentage of WOG. MET source: DL-MET. 0.0 g = Broilers fed diet with 0 g per kg of feed added MET; 0.5 g = Broilers fed diet with 0.5 g per kg of feed added MET; 1 g = Broilers fed diet with 1 g per kg of feed added MET; 2 g = Broilers fed diet with 2 g per kg of feed added MET. ^1^ Data are means of four replicate pens with 10 males and 10 females per pen.

**Table 7:** The effects of added DL-methionine levels on the feed efficiency of the yield parts in kg of feed consumed/kg of broiler parts.

Treatment	WOG (kg/kg)	Wings (kg/kg)	Breast	Breast Fillet (kg/kg)	Tender (kg/kg)	Drumsticks (kg/kg)	Thighs (kg/kg)	Boneless
			Skin (kg/kg)					Thighs (kg/kg)
MET Level								
0.0 g	2.71	34.56	110.60	17.02	71.35	26.81	18.99	24.85
0.5 g	2.59	32.35	106.81	14.58	62.07	25.10	18.75	24.30
1.0 g	2.44	30.46	97.74	13.39	58.38	24.58	17.37	22.72
2.0 g	2.39	29.33	98.23	12.46	57.86	24.56	17.53	22.59

Notes: MET source: DL-MET. 0.0 g = Broilers fed diet with 0 g per kg of feed added MET; 0.5 g = Broilers fed diet with 0.5 g per kg of feed added MET; 1 g = Broilers fed diet with 1 g per kg of feed added MET; 2 g = Broilers fed diet with 2 g per kg of feed added MET. For feed efficiency of the yield parts, kg of feed consumed comes from day 46/48 Feed Intake in Table 3. kg of broiler yield parts is calculated from Equation (2) in Section 2.10 and the day46/68 broiler weights from Table 3 (in kg) and the yield percentages in Table 6 for each part respectively.

**Table 8:** The effects of added DL-methionine levels on the feed efficiency of the yield parts relative to the standard treatment of 1 g MET/kg feed.

Treatment	WOG (%)	Wings (%)	Breast	Breast Fillet (%)	Tender (%)	Drumsticks (%)	Thighs (%)	Boneless
			Skin (%)					Thighs (%)
MET Level								
0.0 g	90%	88%	88%	79%	82%	92%	91%	91%
0.5 g	94%	94%	92%	92%	94%	98%	93%	93%
1.0 g	100%	100%	100%	100%	100%	100%	100%	100%
2.0 g	102%	104%	100%	107%	101%	100%	99%	101%

Notes: MET source: DL-MET. 0.0 g = Broilers fed diet with 0 g per kg of feed added MET; 0.5 g = Broilers fed diet with 0.5 g per kg of feed added MET; 1 g = Broilers fed diet with 1 g per kg of feed added MET; 2 g = Broilers fed diet with 2 g per kg of feed added MET. The feed efficiency of the yield parts relative to standard treatment of 1 g MET/kg feed) is calculated using Equation (3) in Section 2.10. Average feed efficiency of yield part at 1 g MET/kg is taken from Table 7 at 1.0 g for each yield part calculation. Average feed efficiency of each yield part is taken from Table 7. (For example, the percent relative feed efficiency (%) for WOG at 0.0 g TRT = 2.44 kg*100%/2.71 kg = 90.0%).

## Data Availability

The data presented in this study are available within the article.
